# Improving Diagnosis of Depression With XGBOOST Machine Learning Model and a Large Biomarkers Dutch Dataset (*n* = 11,081)

**DOI:** 10.3389/fdata.2020.00015

**Published:** 2020-04-30

**Authors:** Amita Sharma, Willem J. M. I. Verbeke

**Affiliations:** ^1^Department of Operations Research & Quantitative Analysis, Institute of Agri-Business Management, Swami Keshwanand Rajasthan Agricultural University, Bikaner, India; ^2^Erasmus University, Rotterdam, Netherlands

**Keywords:** mental health, classification, extreme gradient boosting, XGBoost, class imbalance, depression

## Abstract

Machine Learning has been on the rise and healthcare is no exception to that. In healthcare, mental health is gaining more and more space. The diagnosis of mental disorders is based upon standardized patient interviews with defined set of questions and scales which is a time consuming and costly process. Our objective was to apply the machine learning model and to evaluate to see if there is predictive power of biomarkers data to enhance the diagnosis of depression cases. In this research paper, we aimed to explore the detection of depression cases among the sample of 11,081 Dutch citizen dataset. Most of the earlier studies have balanced datasets wherein the proportion of healthy cases and unhealthy cases are equal but in our study, the dataset contains only 570 cases of self-reported depression out of 11,081 cases hence it is a class imbalance classification problem. The machine learning model built on imbalance dataset gives predictions biased toward majority class hence the model will always predict the case as no depression case even if it is a case of depression. We used different resampling strategies to address the class imbalance problem. We created multiple samples by under sampling, over sampling, over-under sampling and ROSE sampling techniques to balance the dataset and then, we applied machine learning algorithm “Extreme Gradient Boosting” (XGBoost) on each sample to classify the mental illness cases from healthy cases. The balanced accuracy, precision, recall and F1 score obtained from over-sampling and over-under sampling were more than 0.90.

## Introduction

The 66th General Assembly of the World Health Organization, comprise of Ministers of Health of 194 Member States, adopted the WHO's Comprehensive Mental Health Action Plan 2013–2020^1^ in May 2013. The action plan recognizes the essential role of mental health in achieving health for all. The diagnosis of mental illness is traditionally carried out with interview instruments, clinical judgement and pathological tests. Interview methods are primarily of two types— (a) Interview instruments that are executed by mental health professional, and (b) Patient Self-Reporting instruments.

Due to limitations of interview instruments and clinical judgements, the importance of pathological tests is gaining attention of researchers. The pathological tests supplement the diagnostic decision obtained through interview methods and clinical judgement. The pathological tests are used to measure the biomarker levels in the suspect.

The list of new theories and corresponding biomarkers with potential for predicting depression is expanding (e.g., Strawbridge et al., 2017; Milaneschi et al., 2019). Currently, researchers are taking another research perspective; with the availability of big data sets that contain biomarkers. When exposed to new data, the machine learning based computer programs are able to learn, grow, change, and develop research insights by themselves (e.g., Mitchell, 1997). Researchers are using machine learning techniques to uncover patterns in the data that are largely hypothesis-free, which allows researchers to expand their findings and form innovative theoretical horizons (e.g., Quevedo and Yatham, 2018). There are two approaches to address the complexity in psychiatry disorders—theory driven approach with mechanistic models and agnostic approach. The agnostic approach is based on data driven machine learning and machine learning predictions (e.g., Rutledge et al., 2019).

Machine learning is especially proficient when seeking to explain a variable of interest from a large set of data that not normally distributed. Now a days, governments and agencies are collecting large representative sample data on regular intervals and curating them, e.g., UK Bio Bank (a well-known dataset in Europe), Lifelines Database (a well-known dataset in the Netherlands). This study involves machine learning application for diagnosing the depression cases from healthy cases by using Lifelines Database that contains the biomarkers data and self-reported depression data of Dutch citizens in the Netherlands.

## Literature Review

American Psychiatric Association (2013) defines symptoms (from mild to severe) of Major Depression Disorder (MDD) as follows—

- Feeling sad or having a depressed mood
- Loss of interest or pleasure in activities once enjoyed
- Changes in appetite — weight loss or gain unrelated to dieting
- Trouble sleeping or sleeping too much
- Loss of energy or increased fatigue
- Increase in purposeless physical activity (e.g., hand-wringing or pacing) or slowed movements and speech (actions observable by others)
- Feeling worthless or guilty
- Difficulty thinking, concentrating or making decisions
- Thoughts of death or suicide.

These symptoms should last at least for 2 weeks for Major Depression Disorder. Major Depression Disorder is also called Clinical Depression or Depression. The diagnosis of MDD includes conducting an interview method/instrument, physical examination and in some cases, blood tests.

### Studies Related to Interview Methods for Diagnosing Depression

The studies related to screening of depression subjects from healthy subjects are most widely carried out with the help of interview methods based on interview questionnaires. Center for Epidemiologic Studies Depression Scale 20-item online self-report (CESD; Radloff, 1977), Montgomery-Asberg Depression Rating Scale 10-item diagnostic questionnaire to assess changes with medication (Montgomery and Asberg, 1979), Mood & Feelings Questionnaire for child self-report & parent-report (MFQ; Costello and Angold, 1988), Patient Health Questionnaire 9-item primary care scale for depression severity & treatment monitoring (PHQ9; Löwe et al., 2004), Severity Measure for Depression Adolescents 11–17 years (adapted from PHQ-9 modified for adolescents; Johnson et al., 2002), etc. are most widely used in interview methods for diagnosing the depression.

Diagnostic and Statistical Manual of Mental Disorders (DSM-IV) based depression interviews have long been considered the gold standard for depression diagnosis in research (Löwe et al., 2004). Davison et al. (2009) conducted study of 168 Melbourne aged-care residents with normal cognitive function and found that 27% of depressed residents failed to disclose the symptoms in the clinical interview. Gjerdingen et al. (2009) investigated the effectiveness of DSM-IV based depression interviews because DSM-IV is valued for their diagnostic accuracy and these are often considered to be essential for depression treatment trials. They found that implementing the interview method is problematic due to participant burden. In this study (sample size of 506 mothers of infants), 90% of the women reported some degree of impairment from their depressive symptoms and this keeps results of diagnosis in doubt. Potential problems with a formal depression interview include: increased study costs, need for trained professional in administering the interview, and missed cases.

Pettersson et al. (2015) explored the effectiveness of twenty interview instruments and found only three instruments were meeting the benchmark criteria and they further stated that very few studies scrutinized sensitivity and specificity of interview instruments for diagnosis of depression in clinical research. The structured interview instrument was supported by not more than two studies with a low or moderate risk of bias. This systematic review advised the clinicians that high level of diagnostic accuracy is crucial in clinical practice, and without it, adequate treatment intervention cannot be prescribed. It also constituted the basis for both treatment studies and studies on the etiology, epidemiology and pathophysiology of disease.

Levis et al. (2018) compared the two interview methods—semi structured interview with clinical judgement (CIDI -Composite International Diagnostic Interview) and fully structured interview (MINI—Mini International Neuropsychiatric Interview). Further, they explored that the fully structured interview (MINI) identified more people more depressed than the semi structured interview (CIDI). The studies considered in this review investigation didn't have sample size more than 61 participants with major depression cases based on fully structured interview methods and not more than 22 participants with major depression cases based on semi-structured interview methods. They also found that compared with semi-structured interviews, fully structured interviews tend to classify more people with low level symptoms as depressed, but fewer people with high-level symptoms. This suggests that the choice to use either a fully structured diagnostic interview or a semi-structured interview to classify major depression may influence the diagnosis accuracy.

Due to limitations of the clinical interview methods, doubtful sensitivity and specificity, and high variance in results, it is important to diagnose the association between biomarkers and detecting the depression.

### Studies Related to Biomarkers' Role in Diagnosing Depression

The National Institutes of Health, Biomarkers Definitions Working Group (2001) defined biomarker as “a characteristic that can be objectively measured and evaluated as an indicator of a normal biological process, pathogenic processes, or pharmacologic responses to a therapeutic intervention.”

As the biomarkers are interrelated, the model development becomes very difficult so new methods are required to maximize the consistency and clinical applicability. Standardized and uniform norms for biomarkers have not been widely accepted. Investigating a set of biomarkers simultaneously is an option to inspect isolated markers that could provide a better viewpoint into the complex of biologic systems or networks. Similarly, some authors also recommended the examination of a biomarker panel of several biological factors rather than a single biomarker in the diagnosis of depression and the evaluation of the response to treatment (Schmidt et al., 2011).

Schneider and Prvulovic (2013) reported that to be clinically useful biomarker, method should have high sensitivity and specificity (>80%) in the diagnosis and classification of a disorder. Moreover, for a biomarker to be used in everyday clinical practice, it needs to be reproducible, reliable, inexpensive and non-invasive. Strawbridge et al. (2015) stated that composite biomarker panels are a challenge and opportunity for future research to explore meaningful findings that can be useful to improve treatment outcomes. Hidalgo-Mazzei et al. (2016) emphasized that the use of big data is necessary for resolving the challenges related to heterogeneity, biomarker variability, identifying the optimal markers and bringing the field toward translational, applied research in depression.

Strawbridge et al. (2017) explored that there are primarily two kinds of researches are being conducted to investigate the biomarkers' role in depression—a) The researches focusing on improving the treatment intervention, and b) Detecting the subjects with depression from healthy subjects.

At present there is no approved biomarker as part of the diagnostic criteria for any psychiatric disorder (Bandelow et al., 2017). Currently, the diagnosis of major depressive disorder (MDD) is based on clinical examination and subjective evaluation of depressive symptoms. There is no quantitative test available today for the diagnosis of MDD. Research on biomarkers will be helpful in detecting the disorder and the selection of a treatment, and predicting the response to the treatment. MDD is a clinically and biologically heterogeneous disease, with different clinical appearance and courses of sub-groups, and problems such as the low sensitivity and specificity of the recommended markers reduces the benefit of biomarkers in this disease. The main obstacles in this area may consist of the lack of a suitable animal model of depression, the inclusion of a set of biologically and clinically heterogeneous disorders in MDD, the presence of different subtypes and the continual change of this subgrouping, the high incidence of comorbidities of MDD with many other physical or psychiatric disorders, and the lack of specificity and sensitivity rates of a single biomarker. Many authors have suggested that a wider and multivariate approach could be more useful, including a combination of neuro-imaging, genetic, epigenetic, proteomic and metabolomic approaches (Hacimusalar and Eşel, 2018).

The biomarkers' role can be examined by three types of researches— a) Clinical trial based researches b) Applying Machine Learning/Deep Learning on “behavioral markers” through visual, audio and other form of data, and c) Applying Machine Learning on curated large datasets that contain self-reported depression subject data along with healthy subject data.

### Studies Related to Machine Learning, Biomarkers, and Diagnosing Depression

Alishiri et al. (2008) developed logistic regression model and the variables entered were demographic, clinical, and psychological factors for predicting physical and mental health-related quality of life in rheumatoid arthritis patients. Sensitivity, specificity and accuracy of the physical and mental health were 73.8%, 87%, 83.7%, and 90.38%, 70.36%, 75.43%, respectively on sample of 411 rheumatoid arthritis patients. Mental Health Related Quality of Life was measured by Short Form-36.

Recent advances in deep learning have demonstrated its power to learn and recognize complex non-linear hierarchical patterns based on large-scale empirical data (Bengio et al., 2013). Sacchet et al. (2015) used support vector machine in diagnosing the Major Depression Disorder (MDD) using the neuroimaging. There were 32 participants in the study. Fourteen participants were diagnosed with MDD.

Dipnall et al. (2016) carried out a machine learning boosted regression algorithm and logistic regression study, to identify key biomarkers associated with depression in the National Health and Nutrition Examination Study (NHANES 2009-10). Depression was measured using the Patient Health Questionnaire-9 and 67 biomarkers were analyzed. They used machine learning boosted regression that initially identified 21 biomarkers associated with depression. A final set of three biomarkers were selected. The final three biomarkers from the novel hybrid variable selection methodology were red cell distribution width, serum glucose and total bilirubin. This study included 18 to 80 year old non-institutionalized US civilians (N≈10,000). The final set of 68 binary medical variables and an unweighted sample size of 3,922 was used for clustering in this research. There were 377 participants identified with depression, being representative of the total depressed sample for NHANES 2009-10. The imbalanced nature of the data was dealt with applying clustering algorithm. This research implemented two machine learning algorithms: an unsupervised algorithm, combined with hierarchical clustering, to create the medical symptom clusters and a supervised algorithm to identify and describe the key clusters with a significant relationship with depression.

Sukel (2018), a science writer and author on Managed Healthcare Executive website stated that machine learning is helping to change the mental health in two ways—(i) Identifying the biomarkers; and (ii) Predicting the mental illness cases. Machine learning algorithms can better identify the biomarkers which are relevant to discriminating the mental illness cases and it can help in precision treatment of the mental illness cases.

Zhou et al. (2018) applied support vector machine (SVM) for classification of obsessive-compulsive disorder (OCD) by using whole brain images. In their study, the sample consisted of 48 OCD patients and 45 well-matched health controls.

Victor et al. (2019) developed Artificial Intelligence Mental Evaluation (AiME) and they claim that AiME is capable of detecting depression with minimal human intervention. Furthermore, the researchers claim to ease the challenge of interpreting highly varied physiological and behavioral biomarkers of depression by providing a more objective evaluation. They created a new machine learning based algorithm that leverages, and extends, the behaviorally relevant findings to identify depression using naturalistic audiovisual data. Participants completed the Patient Health Questionnaire (PHQ-9; Kroenke et al., 2001), which is a 9-item self-report measure that assesses depression on a 4-point scale (from 0 = not at all to 3 = nearly every day). They developed a multimodal deep learning model that used video data, audio data, and word content from participants' responses, as well as demographics and other metadata.

Sandhya and Kantesaria (2019) applied logistic regression, k-Nearest Neighbor (*k*NN), Random Forest, Decision Tree, Bagging, Boosting and Neural Network on various sources of data collected from social media platforms like Twitter, Facebook etc. for prediction of mental disorder for employees in IT Industry.

Shatte et al. (2019) reviewed 300 research papers from various databases related to machine learning applications to mental health. They concluded that machine learning applications are applied in the domains of (i) detection and diagnosis; prognosis, (ii) treatment and support; (iii) public health and; (iv) research and clinical administration.

Pandya (2019), Founder of Risk Group & Host of Risk Roundup, wrote about coming computational approach to psychiatry (published on www.forbes.com). The psychiatry has only two sources of information regarding mental illness of a patient i.e., voluntary patient reporting and physician observation based on clinical symptoms or discussions. Most of the psychiatry diagnoses are based only on discussion with the patient. The computational approach to psychiatry with the help of machine learning is expanding horizons of clinical diagnosis in mental health. An article appeared on website—www.medicalfuturist.com. (2019)^2^ stated that Vanderbilt University Medical Center in Nashville uses the various types of data including diagnostic history, gender, age, medication to predict the likelihood of an individual taking suicidal steps. The accuracy of prediction was 84%. The machine learning model was developed on sample of 5,000 patients who were admitted for suicidal steps. The article also mentions that the “smartphone psychiatry movement” started by National Institute of Mental Health. The institute identified 1,000 smartphone based “biomarkers” for detecting the depression.

From the review of literature about machine learning application in mental health, we found that machine learning algorithms are useful in predicting the mental illness cases. In most of the research papers, the sample size was small hence it creates a doubt that the powerful machine learning algorithms like SVM, random forest, *k*NN etc. may overfit the data and will give high variance output when model is applied on new data. Secondly, the dataset taken in these studies were taken from clinical trials in control conditions and the dataset was mostly balanced as categories of outcome had similar proportions. In real life scenario, very rarely the dataset comes with balanced proportions of two classes in target variable.

## Materials and Methods

Here, we apply a research strategy, using Extreme Gradient Boosting (XGBoost)^3^ to identify important biomarkers for depression and predicting the depression cases on different balanced samples obtained from various resampling methods.

This research is based on the Lifelines Cohort study database. Lifelines is a multi-disciplinary prospective population-based cohort study examining, in a unique three-generation design, the health and health-related behaviors of persons living in the north of the Netherlands. It employs a broad range of investigative procedures in assessing the biomedical, socio-demographic, behavioral, physical and psychological factors that contribute to the health and diseases among the general population, with a special focus on multi-morbidity and complex genetics. The cohort profile of the Lifelines study is extensively described by Scholtens et al. (2014).

In this paper, we study depression using an epidemiological study and a large epidemiological data set (*N* = 11,081) from the Lifelines. Prevalence of depression was 5.14% (*n* = 570) in the total study sample. The data set consists of various mental health indicators extracted from self-reports by members from a healthy population (aged 18–89) who volunteered in the Lifelines Project. Here, we focus on biomarkers that the Lifelines has extracted from blood and urine and which are part of the Lifelines standard diagnostic array in profiling a participant's physical health. We focus on a set of 28 biomarkers from a significantly large group of participants from the Lifelines Database that include those related to immune functioning like white blood cells (e.g., neutrophilic granulocytes), red blood cells (e.g., hemoglobin), liver functioning (e.g., creatinine), kidney functioning (urea), or cell metabolism (e.g., calcium). Hence, these are called biomarkers of interest (see Table 1).

**Table 1:** Biomarker variables of interest.

**SN**	**Short name of variable**	**Full name of variable and description**	**Number of subjects in the sample**
1	AF	Alkaline Phosphatase (U/L) (AF is an enzyme found in various tissues and higher concentration is found in liver and bones)	12,597
2	ALB24	Albumin 24 h urine (mg/L) (ALB24 is defined as excretion of albumin per 24 h on 2 of 3 urine collections)	12,546
3	ALT	Alanine Aminotransferase (U/L) (ALT is an enzyme found in liver and kidney)	12,597
4	AST	Aspartate Aminotransferase (U/L) (AST is a blood test used for checking the liver damage)	12,597
5	BA	Basophilic Granulocytes (10E9/L) (These are basically white blood cells)	12,298
6	BALB	Albumin (g/L) (Albumin composes 50%-60% of blood plasma proteins)	12,597
7	BKR	Creatinine (umol/L) (Creatinine is a waste product produced by muscles from the breakdown of a compound called creatine)	12,596
8	CA	Calcium (mmol/L)	12,597
9	CHO	Cholesterol (mmol/L) (Cholesterol is a measure of the total amount of cholesterol in the blood)	12,597
10	EO	Eosinophil Granulocytes (10E9/L) (EO are the white blood cells that releases complex proteins when there is any infection of parasite attack in the body)	12,298
11	ER	Erythrocytes (10E12/L) (ER is red blood cell count)	12,551
12	FOS	Phosphate (mmol/L)	12,597
13	GGT	Gamma-GT (U/L) (GGT stands for gamma-glutamyl transpeptidase or gamma-glutamyl)	12,596
14	GLU	Glucose (mmol/L) (Blood Glucose level)	12,579
15	GR	Neutrophil Granulocytes (10E9/L) (Neutrophils are a type of white blood cell that protect from infections, among other functions)	12,297
16	HB	Hemoglobin (mmol/L) (Hemoglobin is the protein in red blood cells that carries oxygen from the lungs to the body's tissues and returns carbon dioxide from the tissues back to the lungs)	12,551
17	HDC	HDL Cholesterol (mmol/L) (HDL cholesterol removes harmful bad cholesterol. High HDL levels reduce the risk for heart disease – but low levels increase the risk)	12,596
18	HT	Hematocrit (v/v) (It is the ratio of the volume of red blood cells to the total volume of blood)	12,551
19	K	Potassium (mmol/L)	12,594
20	LDC	LDL Cholesterol (mmol/L) (LDL is a low-density lipoprotein. It's bad because it becomes part of plaque, the stuff that can clog arteries and make heart attacks and strokes more likely)	12,597
21	LY	Lymphocytes (10E9/L) (NK cells, T and B cells) (Lymphocytes are white blood cells. They are made in the bone marrow and found in the blood and lymph tissue)	12,297
22	MO	Monocytes (10E9/L) (Monocytes are a type of white blood cell. They are the largest type of leukocytes)	12,297
23	NA	Sodium (mmol/L)	12,584
24	TGL	Triglycerides(mmol/L) (Triglycerides are a fat (lipid) found in blood. The triglycerides are stored in fat cells)	12,597
25	TR	Thrombocytes (10E9/L) (These are platelets that stop bleeding and help wounds to heal)	12,540
26	UKR24	Creatinine 24-h urine (mmol/L) (Creatinine is a waste product produced by muscles from the breakdown of a compound called creatine. Creatinine is removed from the body by the kidneys, which filter almost all of it from the blood and release it into the urine)	12,546
27	UR	Ureum (mmol/L) (Ureum is urea that is a colorless crystalline compound containing nitrogen and this is a product of the breakdown of proteins in the body and is found in urine)	12,597
28	UZ	Uric Acid (mmol/L) (Uric acid is a chemical created when the body breaks down purines)	12,596

*Source: Lifelines Baseline Database. The details about ranges of the biomarkers can be obtained from Lifelines Baseline Database on subscription basis. The researcher cannot share the ranges of biomarker due to restriction distribution condition*.

It is important to understand that a well-generalized classifier for depression cases cannot be possible due to the design and process of the data collection by the Lifelines. However, we assessed the predictive power of biomarkers in diagnosing the depression cases from the sample and found the XGBoost model performed very well in classification tasks on different balanced samples which had equal proportions of both classes (Depression cases and healthy cases).

With the help of machine learning algorithm, cases in the Lifelines database who didn't report the self-reported depression section but they have reported biomarkers can be labeled with the machine learning prediction as a mental illness case or healthy case. This research is going to address and resolve two issues—(i) Machine learning model with high balanced accuracy on imbalanced dataset, and (ii) Providing an alternative and supportive to mental illness diagnosis based on biomarkers.

### Overview of Supervised Machine Learning and Datasets

**Figure 1:**
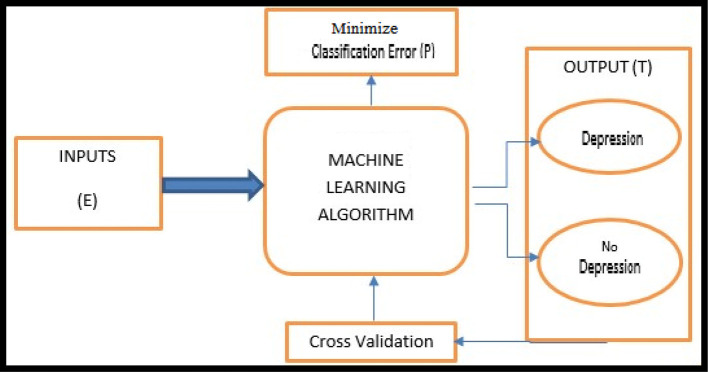
The supervised machine learning model. Source: Authors' own compilation.

A modern definition of Machine Learning is provided by Mitchell (1997, p. 2). The basic machine learning workflow is shown in Figure 1.

As depression in the Lifelines Database is a dependent and dichotomous variable, we used classification supervised learning. More concretely, supervised machine learning algorithms (e.g., Kotsiantis et al., 2007) are applied on datasets that contain a target variable and one or set of predictor variables. The predictors are a set of biomarker measurements (see Table 1) and the target variable is self-reported depression which has two values, 0 and 1, based on the Mini-International Neuropsychiatric Interview (M.I.N.I.), Dutch Version (Sheehan et al., 1998). The respondents were asked two questions: A) “Have you been consistently depressed or down, most of the day, nearly every day for the past 2 weeks?,” the answer “yes” was coded as 1 and “no” as 2; and B) “In the past two weeks, have you been much less interested in most things or much less able to enjoy the things you used to enjoy most of the time?,” where the answer “yes” was again coded as 1 and “no” as 2. We recoded “1” as “1 (yes)” and “2” as “0 (no).” Then we made the following classification: when a person answered “1” to either or both of these questions this was classified as “depressed” and “0” to both questions answered as “not depressed.” Table 1 shows the variables found in the Lifelines database.

There are many studies reported in literature review which used single machine learning model to diagnose the mental illness and very few studies used multiple machine learning models. Instead of applying single machine learning model or multiple machine learning models, in this research, the ensemble machine learning model “XGBoost” is applied. The XGBoost, an ensemble model, initially started as a research project by Chen and Guestrin (2016) as part of the Distributed (Deep) Machine Learning Community (DMLC) group. The “XGBoost” algorithm is a decision tree based algorithm which is very popular in machine learning competitions. Many of the competition winners used XGBoost as their base model to solve machine learning competitions. The XGBoost is a mix of bagging and boosting algorithms which builds weaker learner models initially and improves the learner models accuracy sequentially.

The subjects with depression variable value as “1” in the dataset are the subjects who indicated symptoms of self-reported depression (minority class 5.14% of the sample) and the subjects with depression variable value as “0” in the dataset are the subjects who reported no symptoms of self-reported depression (majority class 94.86% of the sample). These types of datasets pose problem of class imbalance and machine learning algorithms tend to build models biased toward the majority class and it always gives higher accuracy which is misleading. In such cases, not only accuracy but balanced accuracy, precision, recall and F1 score (see Annexure I) measures should also be evaluated for the model performance.

In this research we have used different resampling methods (over-sampling, under-sampling, over-under sampling and ROSE (Random Over-Sampling Examples) sampling) to balance the data and then built the XGBoost model on each sample data. For the convenience, we renamed the resampling methods in following way—over-sampling as O-Sampling, under-sampling as U-Sampling, over-under sampling as OU-Sampling and ROSE sampling as R-Sampling. In O-Sampling method, the minority class observations are increased through duplicating the observations of minority class to be comparable to proportions of majority class observations. In later part of the research paper the sample obtained from O-Sampling is referred as O-Sample. In U-Sampling, the majority class observations are reduced by random procedure and equates the proportion of majority class observations to the proportion of minority class observations. In later part of the research paper, the sample obtained from U-Sampling is referred as U-Sample. In OU-Sampling, the minority class observations are increased through random duplication and majority class observations are reduced through random selection and its result is a sample having equal proportions of both classes. The sample obtained from OU-Sampling is referred as OU-Sample in later part. R-Sampling (Based on ROSE sampling) builds on the generation of new artificial examples from the classes, according to a smoothed bootstrap approach (see, e.g., Efron and Tibshirani, 1997). This is the only reason ROSE sampling is included in spite of already having one oversample from O-Sampling method (O-Sample). The ROSE sample is referred as R-Sample. The original sample is referred as OR-Sample.

The XGBoost model was trained and validated on 80% of the resampled datasets and the prediction from the model was obtained on rest 20% of the resampled test dataset. Five different machine learning models- Xgb.O, Xgb.U, Xgb.OU, Xgb.R, and Xgb.OR were built on O-Sample, U-Sample, OU-Sample, R-Sample and OR-Sample, respectively.

Table 2 shows the proportions of each class of Self-Reported Depression in the balanced samples (O-Sample, U-Sample, OU-Sample and R-Sample) and OR-Sample. Each sample contained all 28 independent variables and target variable.

**Table 2:** Distribution of target variable.

**Sample type**	**Sample size**	**Distribution of target variable**	
		**Healthy subjects = 0**	**Depression subjects = 1**
Original Sample (OR-Sample)	11,081	10,511(94.86%)	570 (5.14%)
Under-Sample (U-Sample)	1,140	570 (50%)	570 (50%)
Over-Sample (O-Sample)	24,000	10,511(44%)	13,489 (56%)
Over-Under-Sample (OU-Sample)	12,000	6,040 (50%)	5,960 (50%)
ROSE Sample (R-Sample)	11,081	5,573 (50%)	5,508 (50%)

*Source: Authors' own computation*.

Mental State Examination that included symptoms of depression and provided us with a total number of relevant cases *N* = 13,395. From these cases, we selected the sample of those who were administered the blood and urine sampling tests with fasting to maintain consistency in the total study sample. Some biomarker variables, such as Apolipoprotein B100 (ApolipoB100 g/L), Free Triiodothyronine (Free T3 pmol/L), Free Thyroxine (Free T4 pmol/L), Apolipoprotein A1 (Apolipo g/L), high-sensitivity C-reactive protein (hs-CRP mg/L), Thyroid Stimulating Hormone (TSH mU/L), had large proportions of missing values hence these variables were removed, which reduced the sample size to *N* = 11,081. Variables such as age and gender were excluded from the computation. The total study sample (*N* = 11,081) consisted of 4,587 male (41.4%) subjects and 6,494 female subjects (58.6%) with a mean age of 48.84 (*SD* = 11.27).

### XGBoost Model Setup

The data analysis was performed on open-source RStudio version 3.5.2. In RStudio, there are various packages that can be installed and called to perform particular statistical analysis tasks. In this research, R packages “xgboost,” “caret,” “mlr,” “ROSE,” “DMwR,” and “ggplot2” were used.

The dataset is divided into three parts—train dataset, validation dataset and test dataset. The train dataset and validation dataset take 80% observations from balanced samples and 20% part of the balanced samples was used as test datasets. The model is first developed on training dataset. After training, model is used for prediction on validation data and the classification errors in prediction on validation dataset is used to fine-tune the model through boosting procedure and this process of training and validation keeps repeating until classification error reduction stops at the specified number of iterations. Final model is obtained from training and validation process. The final model is than used to predict the classification on test dataset. The test data is the dataset which is not shown to the model building process. In model training and validation phase, the model parameters are set to create multiple runs of the model to fine-tune the model performance. Following model parameters were set for model fine-tuning—

$$\begin{array}{l} Booster=“gbtree”,\\ Objective=“binary:logistic”,\\ Eta=0.3,\\ Gamma=0,\\ Max\text{\_}depth=6,\\ Min\text{\_}child\text{\_}weight=1,\\ Subsample=1,\\ Colsample\text{\_}bytree=1 \end{array}$$

Each element of XGBoost model set up is separately explained in the Annexure I.

XGBoost model named as “Xgb” is trained on train data and validated on validation dataset of resampled datasets—

(1) $$\begin{array}{c} xgb=xgb.train\;(params=params,\;data=dtrain,\;nrounds\\ =\;100,\;watchlist=list\;\left(val=dval,\;train=dtrain\right),\;nfold\\ =\;5,\;showsd=TRUE,\;stratified=TRUE,\;print\text{\_}every\text{\_}n\\ =10,\;early\text{\_}stopping\text{\_}rounds=20,\;maximize=F,\\ eval\text{\_}metric=“error”) \end{array}$$

The elements of XGBoost model training parameters are separately explained in the Annexure I.

XGBoost models- Xgb.O, Xgb.U, Xgb.OU, Xgb.R, and Xgb.OR are built on O-Sample, U-Sample, OU-Sample, R-Sample, and OR-Sample datasets, respectively. These models are used to do prediction on test datasets. The predictions obtained from each model is evaluated through confusion matrix.

## Results

The XGBoost models- Xgb.O, Xgb.U, Xgb.OU, Xgb.R, and Xgb.OR were developed on O-Sample, U-Sample, OU-Sample, R-Sample and OR-Sample, respectively. All measures of confusion matrix are calculated and the results obtained are shown in Table 3.

**Table 3:** Measures of confusion matrix.

**Confusion matrix elements**	**Original dataset (OR-Sample)**	**Under-sample dataset (U-sample)**	**Over-sample dataset (O-sample)**	**Over-under sample dataset (OU-sample)**	**ROSE sample dataset (R-sample)**
Accuracy	0.9035	0.5164	0.9729	0.9442	0.6618
95% CI	0.8949, 0.9115	0.4724, 0.5603	0.9696, 0.9758	0.9378, 0.9502	0.6485, 0.6749
No information rate	0.9488	0.5551	0.5838	0.5334	0.5107
p-value [Acc>NIR]	1.000	0.96495	<2.2e-16	<2.2e-16	<2.2e-16
Specificity	0.10156	0.5017	0.9982	0.9825	0.6669
Balanced accuracy	0.52413	0.5183	0.9765	0.9466	0.6617
Precision	0.0932	0.5017	0.9548	0.9107	0.6565
Recall	0.10	0.5737	0.9987	0.9835	0.6538
F1	0.0972	0.5183	0.9762	0.9457	0.6551

*Source: Authors' own computation*.

The Balanced Accuracy, Precision, Recall and F1 Score are calculated with the help of confusion matrix. Confusion matrix^4^ is a 2X2 contingency table explained in Annexure I.

When dataset is balanced overall accuracy is sufficient to evaluate a classification machine learning model but in this research the dataset is highly imbalanced. It is highly recommended to observe other performance values to evaluate model classification and that is the reason other performance values like Balanced Accuracy, Precision, Recall and F1 Score are calculated. Precise definitions and formula of Balanced Accuracy, Precision, Recall and F1 Score are described in Annexure I.

The Table 4 shows that the best performance of the XGBoost model- Xgb.O is obtained on test dataset obtained from O-Sample with highest measures in Accuracy (0.9729), Balanced Accuracy (0.9765), Precision (0.9548), Recall (0.9987), and F1 Score (0.9762).

**Table 4:** Variable importance extracted from different XGBoost models.

**Sr. no**.	**Xgb.OR**	**OR.Gain**	**Xgb.U**	**U.Gain**	**Xgb.O**	**O.Gain**	**Xgb.OU**	**OU.Gain**	**Xgb.R**	**R.Gain**
1	GR	0.06	FOS	0.07	TR	0.06	TGL	0.07	GGT	0.11
2	UZ	0.06	LY	0.06	TGL	0.05	GR	0.06	ALB24	0.10
3	FOS	0.06	LDC	0.05	AF	0.05	LY	0.05	TGL	0.05
4	TGL	0.05	CA	0.05	UKR24	0.05	TR	0.05	GLU	0.05
5	CHO	0.05	BALB	0.05	GR	0.05	UKR24	0.05	AF	0.04
6	UR	0.05	TGL	0.05	ER	0.05	UR	0.05	HDC	0.04
7	MO	0.05	MO	0.05	LY	0.05	AF	0.05	LDC	0.04
8	TR	0.04	UZ	0.05	UR	0.05	FOS	0.04	FOS	0.03
9	BKR	0.04	TR	0.04	FOS	0.05	GLU	0.04	GR	0.03
10	LY	0.04	AF	0.04	CA	0.04	UZ	0.04	BA	0.03
11	UKR24	0.04	UR	0.04	ALB24	0.04	HT	0.04	UKR24	0.03
12	CA	0.04	GR	0.04	ALT	0.04	BKR	0.04	UR	0.03
13	ER	0.04	CHO	0.04	GGT	0.04	ER	0.04	NA.	0.03
14	EO	0.04	UKR24	0.03	GLU	0.04	ALB24	0.04	HB	0.03
15	ALB24	0.04	K	0.03	HT	0.03	CA	0.03	AST	0.03
16	AF	0.03	BKR	0.03	EO	0.03	ALT	0.03	MO	0.03
17	HB	0.03	ER	0.03	UZ	0.03	EO	0.03	BALB	0.03
18	HT	0.03	AST	0.03	CHO	0.03	CHO	0.03	BKR	0.03
19	LDC	0.03	GLU	0.03	BKR	0.03	AST	0.03	LY	0.03
20	GLU	0.03	ALT	0.03	MO	0.03	LDC	0.03	UZ	0.03
21	AST	0.03	HT	0.03	BALB	0.03	BALB	0.03	ALT	0.03
22	GGT	0.03	HDC	0.02	K	0.03	GGT	0.02	HT	0.03
23	ALT	0.02	ALB24	0.02	AST	0.03	MO	0.02	CA	0.03
24	K	0.02	HB	0.02	HDC	0.03	HDC	0.02	EO	0.03
25	HDC	0.02	BA	0.02	LDC	0.02	K	0.02	TR	0.02
26	BALB	0.02	GGT	0.01	HB	0.02	HB	0.02	CHO	0.02
27	BA	0.01	EO	0.01	NA.	0.01	BA	0.02	K	0.02
28	NA.	0.01	NA.	0.01	BA	0.01	NA.	0.01	ER	0.02

*Source: Authors' own computation*.

To know how each predictor variable is contributing to the accuracy of the model, variable importance is calculated. The variable importance shows that how a variable improves the accuracy and this is measured by Gain. Gain is the improvement in accuracy brought by a variable on the branch it is on the decision tree. If addition of any feature improves the classification accuracy or reduces classification error, the higher Gain score is given to the variable. In Table 4, each sample's feature list (Xgb.O, Xgb.U, Xgb.OU, Xgb.R, and Xgb.OR) and their corresponding Gain scores (O.Gain, U.Gain, OU.Gain, R.Gain, and OR.Gain) are given.

The variable importance from Xgb.O and Xgb.OU models are plausible as the Balanced Accuracy, Precision, Recall and F1 Score is the highest.

It is noteworthy that the hypothesis test result for “No Information Rate” (see Annexure I) is significant at 0.05 alpha level for both the models (Xgb.O and Xgb.OU). This indicates that the overall accuracy rate of the model is greater than the accuracy rate of the majority class in the target variable.

## Discussion

This study was set to explore the predictability of biomarkers in diagnosing and classifying the depression cases from healthy cases through XGBoost algorithm. According to the Xgb.O model, the variables – Thrombocytes (10E9/L), Triglycerides (mmol/L), Alkaline Phosphatase (U/L), Creatinine 24-h urine (mmol/L) and Neutrophil Granulocytes (10E9/L) have highest variable importance and the variables– HDL Cholesterol (mmol/L), LDL Cholesterol (mmol/L), Hemoglobin (mmol/L), Sodium (mmol/L) and Basophilic Granulocytes (10E9/L) have the lowest variable importance in the model. According to the Xgb.OU model, the variables—Triglycerides (mmol/L), Neutrophil Granulocytes (10E9/L), Lymphocytes (10E9/L), Thrombocytes (10E9/L) and Creatinine 24-h urine (mmol/L) have the highest variable importance and the variables— HDL Cholesterol (mmol/L), Potassium (mmol/L), Hemoglobin (mmol/L), Basophilic Granulocytes (10E9/L), and Sodium (mmol/L) have the lowest variable importance in the model.

We are convinced that these conclusions are robust. First, due to the fact that machine learning allows the use of many different biomarkers and variable importance hierarchy, it allows detection of depression through many variables that could indicate dysfunction of various physiological processes occurring in different organs and tissues, as these variables compete for magnitude in predicting depression. Second, we used very robust and popular algorithm Extreme Gradient Boosting (XGBoost) machine learning technique in order to secure the predictive validity of the biomarkers.

Our results highlight the benefits of XGBoost model with O-Sample dataset and OU-Sample datasets for classifying the depression cases from healthy cases on the Lifelines database sample of 11,081 Dutch citizens. As XGBoost is computationally less expensive than the Neural Networks, it is better alternative for implementation and secondly, the neural networks involve complex calculations and it is not easy to interpret the model development process in neural network model setup. The XGBoost is not only computationally less expensive but it also does not require rich data like medical imaging data and it is easy to interpret in comparison to Neural Net based models. The XGBoost is also a better modeling process in comparison to the use of single machine learning models like Logistic Regression, Support Vector Machine, Decision Tree etc. The cross validation of the model is very robust and inbuilt in the XGBoost model as the XGBoost is an ensemble modeling wherein multiple models are built sequentially to reduce classification error on each iteration.

In this study, we researched the relationship between a set of biomarkers and self-reported depression, using XGBoost machine learning technique. Our robust finding on the relationship between biomarkers as indicators of depression might help mental health practitioners in two different ways. First, in order to diagnose people who are possibly suffering from depression, practitioners may send their patients to a medical center where their blood and urine is collected and subsequently sent to a laboratory for testing. The data of lab test can be fed into XGB Model to predict the outcome (Depression or healthy case). The method could be useful when there are cases of impairments due to depression and patient is not responding to the interview methods or there is doubtful outcome derived from interview method.

## Limitations and Future Research

Note that the measured indicators of depression were rather simple and do not reflect the complete questionnaire of the Mini-International Neuropsychiatric Interview, Dutch Version (Sheehan et al., 1998). Using this questionnaire and stressing on completely filling the questionnaire would have improved the measuring process, but this would have put too much of a strain on the work load of the medical staff at the Lifelines. As this research used database of Dutch citizens only, the research has limitations of generalization for other groups, nationalities, etc. In light of the fact that there are no agreed and accepted standards for biomarker levels across different countries and ethnic groups, the XGBoost model developed in this research cannot be applied for other countries and ethnic group, a fresh model building process should be used. As this research was focused only on diagnosing the depression cases and not aimed at diagnosing the type of depression, in future researches, machine learning should be applied to explore precisely the types of depression.

Note that our study, for instance, did not include genetic or epigenetic markers. Nor did we include cytokines, as they are not part of the Lifelines database. Future research could, of course, include these markers. This study, although robust given the XGBoost models with different samples and the amount of data used, should be replicated in clinical trial settings.

## Data Availability Statement

The datasets presented in this article are not readily available because it is obtained from Lifelines, who provide access to data through secured cloud access after obtaining due permissions and fee payment for a limited time period. Requests to access the datasets should be directed to https://www.lifelines.nl/researcher.

## Author Contributions

WV: supervised the research work and facilitated the data access from Lifelines and conceptualized the exploration of association with biomarkers and depression through machine learning models. AS: worked on applications of machine learning on the Lifelines dataset and frame the research design and writing of the research paper.

## Conflict of Interest

The authors declare that the research was conducted in the absence of any commercial or financial relationships that could be construed as a potential conflict of interest.

## Footnotes

^1^https://www.who.int/mental_health/action_plan_2013/en/ (Retrieved October 1, 2019).

^2^https://medicalfuturist.com/artificial-intelligence-in-mental-health-care/ (Retrieved on October 16, 2019).

^3^Beginners Tutorial on XGBoost and Parameter Tuning in R. Available online at: https://www.hackerearth.com/practice/machine-learning/machine-learning-algorithms/beginners-tutorial-on-xgboost-parameter-tuning-r/tutorial/ (accessed December 1, 2019).

^4^*Confusion Matrix*. Retrieved from: https://towardsdatascience.com/understanding-confusion-matrix-a9ad42dcfd62 (accessed December 1, 2019).
