# Supplementary material for: Improving Diagnosis of Depression With XGBOOST Machine Learning Model and a Large Biomarkers Dutch Dataset (n = 11,081)
Source: Front Big Data. 2020 Apr 30;3:15. doi: 10.3389/fdata.2020.00015 (PMC7931945; doi:10.3389/fdata.2020.00015)
Supplement: Supplementary file 1 [file Data_Sheet_1.pdf]

Annexure I

1. XGBoost Model Setup

The XGBoost model is a decision tree boosting algorithm and it is developed by Chen and Guestrin (2016). The Booster parameter defines the type of booster. The “gbtree” and “gblinear” are two boosters available in XGBoost algorithm. As the outcome variable in this research is binary, the “gbtree” (a decision tree based classification algorithm) was used. The booster improves the model successively by learning from misclassification and fine-tunes the model.

The “Objective” parameter in XGBoost defines the machine learning task to be performed. There are many options available in “Objective” parameter like “reg:squarederror”, “reg:squaredlogerror”, “reg:logistic”, “binary:logistic”, “binary:logitraw”, “binary:hinge”, “count:poisson”, “survival:cox”, “multi:softmax”, “multi:softprob”, “rank:pairwise”, “rank:ndcg”, “rank:map”, “reg:gamma” and “reg:tweedie”. The “binary: logistic” objective parameter was chosen as the target variable for the research is a dichotomous variable (depression Vs. healthy subject).

The “Eta” parameter in XGBoost algorithm is also called the Learning Rate. In this research, “Eta” is set at default value 0.3. Eta shrinks the weights of the features after each boosting step. The “Gamma” parameter of XGBoost algorithm is the minimum loss reduction required to make further partition on a leaf node of a tree. Its value ranges from 0 to infinity. In this research, “Gamma” parameter is set to zero. The “Max_depth” parameter in XGBoost algorithm defines the depth of a decision tree. It was set at default value 6 which means that the maximum depth of decision tree will be upto 6 nodes maximum. The “Min_Child_Weight” parameter in XGBoost algorithm defines the minimum leaf node to be used for further partitioning. “Sub_Sample” parameter in XGBoost algorithm defines

proportion of sample goes into each iteration of model building. It ranges between 0 and 1. The default value 1 is selected for "Sub_Sample" parameter. "Colsample_Bytree" parameter in XGBoost defines the number of feature variables go into model building in each iteration. The default value 1 is chosen for "Colsample_Bytree" parameter.

2. XGBoost Model Training Setup

After XGBoost model setup, the model training setup is defined. In XGBoost model, there are various parameters which can be set for robust training of the model. Following XGBoost model training setup was used in the research---

*xgb=**xgb.train**(params=params, data=dtrain, nrounds=100, watchlist = **list**(val=dval, train=dtrain), nfold = 5, showsd=TRUE, stratified = TRUE, print_every_n = 10, early_stopping_rounds = 20, maximize = F, eval_metric="error") ……….Eq.(1)*

After selecting the XGBoost model setup parameters, the model training parameters were set to execute XGBoost model building. Here, "params" are the set of parameters defined in the XGBoost model setup. Data indicates the training dataset (named as dtrain). The "nrounds" parameter defines the number of times the model building process will be repeated. Here, the "nrounds" is set at 100. The "watchlist" parameter tells to evaluate the model predictions on two datasets—validation dataset (dval) and train dataset (dtrain). The training dataset is divided into two sets—validation dataset (dval) and train dataset (dtrain) by 5 fold cross validation on each iteration. The "showsd" parameter is set as "TRUE". The "eval_metric" parameter is set to "error" which tells the classification error of the final model(https://www.hackerearth.com/practice/machine-learning/machine-learning-algorithms/beginners-tutorial-on-xgboost-parameter-tuning-r/tutorial/).

3. Confusion Matrix

Confusion Matrix is the primary evaluation matrix for classification machine learning models.

It is a 2X2 contingency table as show in the following figure---

		Actual Values	
		Positive (1)	Negative (0)
Predicted Values	Positive (1)	TP	FP
	Negative (0)	FN	TN

Source: https://towardsdatascience.com/understanding-confusion-matrix-a9ad42dcfd62

Reproduced from Narkhede S. with permission

The columns in confusion matrix represent the actual values of dependent variable from the test dataset and the rows represent the predicted values obtained from the machine learning model on test dataset. TP is the True Positives which tells that the observations which are positive in test dataset and are also predicted positive by the Machine Learning model. TN is the True Negatives which tells that the observations which are negative in test dataset and are also predicted negative by the Machine Learning model.

FP is the False Positives which tells that the observations which are positive in test dataset and are wrongly predicted as negative by the Machine Learning model. FN is the False Negatives which tells that the observations which are negative in test dataset and are wrongly predicted as positive by the Machine Learning model.

Based on TP, TN, FP and FN, various measures of classification accuracy are calculated. Formula for calculating various measures of classification accuracy are as follows---

Accuracy = [(TP + TN) / (TP + TN + FP + FN)]

Formula (1)

Accuracy is basically overall accuracy. It is a ratio of total correctly classified observations divided by total number of observations. Desired value of Accuracy for a classification model is near to 1.

Precision = TP / (TP + FP)

Formula (2)

Precision is the ratio of number of True Positive observations and total True Positive observations. Desired value of Precision for a classification model is near to 1.

Recall = TP / (TP + FN)
Formula (3)

Recall is defined as the ratio of the total number of correctly classified positive classes and the total number of positive classes. Desired value of Recall for a classification model is near to 1.

F1 Score = 2 * (Precision * Recall) / (Precision + Recall)
Formula (4)

It is difficult to compare two models with different Precision and Recall. So to make them comparable, F-Score is used. It is the Harmonic Mean of Precision and Recall. As compared to Arithmetic Mean, Harmonic Mean punishes the extreme values more. F-score should be high.

Balanced Accuracy = [{ TP / (TP + FP) } + { TN / (TN + FN) }]
Formula (5)

Balanced Accuracy is useful when dataset is imbalanced. It shows better picture of classification accuracy. Ideally, it should be near to 1.

Specificity = TN / (TN + FN)

Specificity determines the proportion of actual negatives that are correctly identified. It should also be high.

The "No-Information Rate" is the largest proportion of the observed classes (there are more class 2 data than class 1 in the test set). A hypothesis test is also computed to evaluate whether the overall accuracy rate of machine learning model predictions is greater than the rate of the largest class. If p-value for "No-Information Rate" is less than 0.05

significance value, the null hypothesis is rejected and alternative hypothesis “model accuracy is greater than the rate of largest class” is accepted.
